# Database analysis of patients with hepatocellular carcinoma and treatment flow in early and advanced stages

**DOI:** 10.1002/prp2.486

**Published:** 2019-06-20

**Authors:** Keishi Akada, Noriyuki Koyama, Shigeru Taniguchi, Yuji Miura, Ken Aoshima

**Affiliations:** ^1^ Eisai Co. Ltd. Tokyo Japan

**Keywords:** chemotherapy, database, hepatocellular carcinoma, therapeutic chemoembolization

## Abstract

Despite recent developments in treatment modalities and diagnosis, the prognosis of advanced hepatocellular carcinoma (HCC) remains unsatisfactory. To gain insight into treatment decisions for HCC patients, their characteristics and treatment flow in the early and advanced stages were examined. HCC patients' characteristics and treatment flow were retrospectively analyzed using the Japanese medical claims database. The 8999 patients' mean age at HCC diagnosis was 71.1 years, with no difference between early (Stage I/II) and advanced (Stage III/IV) stages. The mean observation period was 26.2 months, shorter in advanced than in early stages. HCV hepatitis was reported in 52.0% of HCC patients, with concomitant hypertension in 53.4%, type 2 diabetes in 45.8%, cirrhosis in 39.3%, and hyperlipidemia in 15.5%. The rates of HCV hepatitis, hypertension, and hyperlipidemia decreased with stage progression. Analysis of treatment flow showed that, at all disease stages, transcatheter arterial chemoembolization (TACE) was the most common first to fourth‐line treatment. Epirubicin was the most frequently (44.1%) used chemotherapeutic agent for first‐line TACE, followed by miriplatin (23.6%) and cisplatin (12.3%). With stage progression, cisplatin use increased. Sorafenib was used concomitantly for first‐line TACE in 3.2% of patients, and its use increased significantly in advanced stages. Clear differences in baseline characteristics and treatment flow between early and advanced stages were identified. Continuous analysis of the database with longer follow‐up may provide useful information about treatment selection and prediction of outcome such as survival.

**Abbreviations**
BCLC: Barcelona Clinic Liver Cancer
HAIC: hepatic arterial infusion chemotherapy
HBV: hepatitis B virus
HCC: hepatocellular carcinoma
HCV: hepatitis C virus
RFA: radiofrequency ablation
TACE: transcatheter arterial chemoembolization

## 1 INTRODUCTION

Hepatocellular carcinoma (HCC) is one of the most common cancers worldwide; its primary risk factors include chronic infection by hepatitis B virus (HBV) or hepatitis C virus (HCV). Treatment options for HCC include resection, local ablation, hepatic arterial infusion chemotherapy (HAIC), transcatheter arterial chemoembolization (TACE), and liver transplantation.^1^, ^2^ Advances in treatment modalities and diagnosis have considerably improved the overall survival rate for HCC patients.^3^, ^4^ As the surveillance rate for HCC patients has increased in Japan, the disease stage at diagnosis has decreased, and the survival rate has concomitantly improved.^5^ However, despite the developments in treatment modalities and diagnosis, the prognosis of advanced HCC patients remains unsatisfactory. Patients often experience recurrence, with limited treatment options for advanced stages of the disease.

Choice of treatment flow may differ with HCC stage.^6^, ^7^ According to the Barcelona Clinic Liver Cancer (BCLC) staging system, curative therapies such as liver transplantation, liver resection, and radiofrequency ablation (RFA) are recommended in early‐stage HCC. TACE is widely used when curative therapies cannot be performed, and it is recommended for intermediate‐stage HCC and as a palliative treatment in advanced‐stage HCC.^6^, ^7^ TACE can be an option in early‐stage HCC for reasons such as poor residual liver function, comorbidities for surgery, and difficult RFA treatment location.^6^, ^7^ Sorafenib is recommended in advanced‐stage HCC.^6^

Because a large claim database analysis can reflect real‐world medical circumstances, evidence has been accumulated through database research. Japan has a nation‐wide health coverage system in which all citizens can receive health insurance and treatment. The national database that registers medical claims by all health care insurance has been developed and became available for research. In a recent analysis in Japanese claims database, patients with liver disease related to HBV/HCV infection showed a higher incidence of HCC when aged ≥ 60 years.^8^ In 4713 patients with liver cirrhosis or HBV/HCV infection, the HCC surveillance rate during follow‐up was higher for patients with HBV/HCV infection than for those with nonviral cirrhosis.^9^

In this study, the baseline characteristics and treatment flow of HCC patients were analyzed using the Japanese claims database. The common therapies for HCC patients in early and advanced stages were identified, and the impact of tumor stage progression on the choice of treatment was examined.

## 2 METHODS

### 2.1 Study design and data source

This epidemiological study was conducted in accordance with the Guidelines for Good Pharmacoepidemiology Practices,^10^ with particular attention to the differences in the characteristics of HCC patients and in treatment flow between early and advanced stages of the disease.

The Japanese medical claims database provided by Medical Data Vision Co., Ltd (MDV; Tokyo, Japan) was used for this study; it contains hospitalization summary, laboratory result, disease history, and medical claims data. The database source population was derived from 314 hospitals in Japan using the Diagnosis Procedure Combination system; the number of patients was approximately 20 million on March 31, 2017.^11^ The database contains: an anonymized patient identifier; sex; age; medical service date; disease history; drug treatment; laboratory value standard set; and hospitalization data, comprising the outcome, cancer stage, Child‐Pugh score, and other data related to patients' conditions. Age and sex distributions of HCC patients in this database are approximately similar to those in the National Database of Health Insurance Claim Information and Specified Medical Checkups, Japan.^12^ In this study, the HCC patient data collected from 1 April 2008 to 31 January 2017 were analyzed.

### 2.2 Disease definitions

According to the International Statistical Classification of Diseases: 10th Revision (ICD‐10), HCC was identified with a definitive diagnosis of C220 meaning liver cell carcinoma. A history of ordering α‐fetoprotein (AFP) and des‐γ‐carboxy prothrombin (DCP), diagnostic markers for HCC, was the second criterion for confirming HCC.

To understand HCC disease comorbidity, relevant comorbid diseases were defined using ICD‐10 codes. Type 2 diabetes mellitus (DM) was identified by E11 (noninsulin‐dependent DM), E12 (malnutrition‐related DM), E13 (other specified DM), and E14 (unspecified DM); hyperlipidemia by E78 (disorders of lipoprotein metabolism and other lipidaemias); hypertension by I10 (essential/primary hypertension), I11 (hypertensive heart disease), I12 (hypertensive renal disease), I13 (hypertensive heart and renal disease), and I15 (secondary hypertension); cirrhosis by K703 (alcoholic cirrhosis of liver), K743 (primary biliary cirrhosis), K744 (secondary biliary cirrhosis), K745 (biliary cirrhosis, unspecified), and K746 (other and unspecified cirrhosis of liver); HBV by B16 (acute hepatitis B) and B181 (chronic viral hepatitis B); HCV by B171 (acute hepatitis C) and B182 (chronic viral hepatitis C); and nonviral hepatitis by K701 (alcoholic hepatitis), K711‐K716 (toxic liver disease), K720 (hepatic failure, not elsewhere classified), K730/K732/K738/K739 (chronic hepatitis, not elsewhere classified), K754 (autoimmune hepatitis), K758 (other specified inflammatory liver diseases), K759 (inflammatory liver disease, unspecified), and K760 (fatty (change in) liver, not elsewhere classified).

### 2.3 Treatment definitions

Criteria for identifying treatment onset in the Japanese medical claims database were defined according to the treatment category codes used for treatment reimbursement in Japan. RFA was identified with codes starting with “K697,” HAIC with those starting with “K611,” percutaneous ethanol injection (PEI) with those starting with “J017,” hepatectomy with those starting with “K695,” and transcatheter arterial embolization (TAE) with those starting with “K615/K6151/K6152/K6153.” When there was a prescription history of anticancer drugs on the day of TAE, treatment was defined as TACE.

In the MDV database, the anatomical therapeutic chemical classification (ATC) code, defined by the European Pharmaceutical Market Research Association, was provided to classify the drugs. The anticancer drugs used as chemotherapeutic agents for TACE were identified using ATC codes starting with “L01,” but tablet‐type drugs were excluded. Sorafenib (ATC code “L01H0”) was a standard drug for HCC.

### 2.4 Study design and population

The study aimed to understand the baseline characteristics of HCC patients and the pattern of treatment flow in the real‐world. Patient data were extracted from the MDV database with the following conditions: having a definitive HCC diagnosis with an order history of AFP/DCP (HCC diagnostic markers); at least one treatment conducted using TACE, TAE, hepatectomy, RFA, PEI, HAIC, or sorafenib chemotherapy with > 180 days of follow‐up after definitive diagnosis; and primary HCC patients with cancer stage data registered. The last two conditions helped discriminating HCC from other tumor patients. In total, 71,947 patients initially visited hospitals from 1 April 2008 to 31 January 2017, and the data of 8999 patients satisfying the above conditions were extracted for the analysis of patient characteristics and treatment flow.

### 2.5 Analysis of patient characteristics and treatment flow

Body mass index (BMI), cancer stage, Child‐Pugh score, and serum laboratory values were extracted from the data between the admission date for the initial hospitalization with definitive diagnosis and the HCC treatment initiation date. Comorbidity and the Charlson comorbidity index (CCI) were calculated from the data before the discharge date for the initial hospitalization with a definitive diagnosis. The definition of the observation period began on the earliest admission date with a definitive diagnosis of primary HCC and ended on the last prescription date.

Treatment flow for patients was visualized using a Sankey diagram.^13^ The rates of initial treatments were determined from prescription orders, focusing on treatments including TACE, TAE, RFA, hepatectomy, PEI, HAIC, and sorafenib chemotherapy.

Statistical analysis was performed using R 3.4.1. Student's *t* test, Wilcoxon's rank‐sum test for continuous variables, and Fisher's exact test for categorical variables were used to assess differences between early and advanced stages.

## 3 RESULTS

A total of 8999 patients were enrolled in the analysis of the characteristics of HCC patients from the Japanese medical claims database; 6594 were in the early stages (Stage I/II), and 2405 were in the advanced stages (Stage III/IV). The patients' characteristics are summarized in Table 1. The mean age at HCC diagnosis was 71.1 years, with no difference between early and advanced stage patients. The observation period decreased with tumor stage progression. The number of males was significantly higher than the number of females in both groups and the number of males increased with tumor stage progression. BMI did not differ between stages. In the analysis of comorbid diseases, there was a high rate of HCC patients with HCV hepatitis, followed by HBV hepatitis or nonviral hepatitis. HCV hepatitis was reported in 52.0% of HCC patients, with concomitant hypertension in 53.4%, type 2 diabetes in 45.8%, cirrhosis in 39.3%, and hyperlipidemia in 15.5%. The rates of HCV hepatitis, hypertension, and hyperlipidemia were lower in advanced stage patients than early stage. The rates of type 2 diabetes mellitus, cirrhosis and CCI did not differ between stages.

**Table 1:** Characteristics of patients with hepatocellular carcinoma

	All stages (N = 8999)	Early stages (N = 6594)	Advanced stages (N = 2405)	*P*
Age at HCC diagnosis (y)	71.1 ± 9.5	71.2 ± 9.4	71.0 ± 9.5	0.492
Observation period (months)	26.2 ± 16.5	27.4 ± 16.8	22.9 ± 15.2	<0.001
Male sex (%)	69.2%	66.1%	77.9%	<0.001
BMI	23.8 ± 6.3	23.8 ± 6.9	23.8 ± 3.8	0.962
Hepatitis				
HCV	52.0%	55.2%	43.0%	<0.001
HBV	19.4%	18.9%	20.8%	0.050
Nonviral	16.0%	16.2%	15.6%	0.455
Hypertension	53.4%	54.1%	51.5%	0.029
Type 2 DM	45.8%	45.9%	45.7%	0.867
Cirrhosis	39.3%	39.3%	39.5%	0.883
Hyperlipidemia	15.5%	16.0%	14.0%	0.022
CCI	4.9 ± 2.9	4.9 ± 2.9	4.9 ± 2.7	0.519

Means ± standard deviations are shown for continuous variables. The differences in mean values between early and advanced stages were assessed, and *P*‐values were calculated using the *t* test, Wilcoxon rank‐sum test, or Fisher's exact test. Because of the missing BMI values, 8746 patients in all stages, 6423 patients in early stages, and 2323 patients in advanced stages were analyzed. HCC, hepatocellular carcinoma; BMI, body mass index; DM, diabetes mellitus; CCI, Charlson comorbidity index.

Serum laboratory values and Child Pugh scores were analyzed in a limited number of patients (Table 2). For example, the value of platelet count was available in only 815 patients and Child Pugh score was in 2298 patients among total 8999 patients. Although the platelet count, ALP, and γ‐GTP values were higher in advanced stage patients than in early stage patients, other liver function markers such as albumin, AST, ALT, and LDH did not show significant differences between stages. The proportions of Child Pugh score A and Child Pugh score B or C were also similar.

**Table 2:** Serum laboratory values and Child Pugh scores

	All stages	*N*	Early stages	*N*	Advanced stages	*N*	*P*
Platelet count (×10 000/μL)	11.1 (8.2–15.8)	815	10.6 (8.0–14.9)	569	12.2 (9.1–17.8)	246	<0.001
PT INR	1.1 (1.1–1.2)	693	1.1 (1.1–1.2)	474	1.1 (1.1–1.2)	219	0.982
Albumin (g/dL)	3.4 (2.9–3.8)	799	3.3 (2.9–3.8)	558	3.4 (3.0–3.8)	241	0.380
AST (U/L)	55 (36–104)	806	56 (35–108)	562	54 (38–84)	244	0.582
ALT (U/L)	44 (26–86)	805	45 (26–89)	561	41 (26–79)	244	0.572
LDH (U/L)	222 (185–282)	787	222 (185–278)	545	221 (186–284)	242	0.726
ALP (U/L)	296 (215–402)	773	291 (214–381)	536	317 (216–447)	237	0.028
γ‐GTP (U/L)	51 (29–100)	767	44 (27–86)	530	66 (38–129)	237	<0.001
Triglycerides (mg/dL)	94 (68–124)	259	95 (68–131)	180	94 (67–120)	79	0.295
Total cholesterol (mg/dL)	153 (133–176)	314	153 (134–174)	213	154 (131–183)	101	0.498
Total bilirubin (mg/dL)	0.9 (0.6–1.2)	796	0.9 (0.6–1.2)	554	0.9 (0.6–1.3)	242	0.639
Direct bilirubin (mg/dL)	0.2 (0.1–0.4)	462	0.2 (0.1–0.4)	314	0.3 (0.2–0.4)	148	0.234
Child Pugh A	61.0%	2298	61.5%	1646	59.5%	652	0.368
Child Pugh B/C	39.0%		38.5%		40.5%		

Medians (interquartile range) are shown for skewed continuous variables. The differences in mean values between early and advanced stages were assessed and *P*‐values were calculated using the *t* test, Wilcoxon rank‐sum test, or Fisher's exact test. PT INR, prothrombin time‐international normalized ratio; AST, aspartate aminotransferase; ALT, alanine aminotransferase; LDH, lactate dehydrogenase; ALP, alkaline phosphatase; γ‐GTP, γ‐glutamyl transpeptidase.

**Figure 1:**
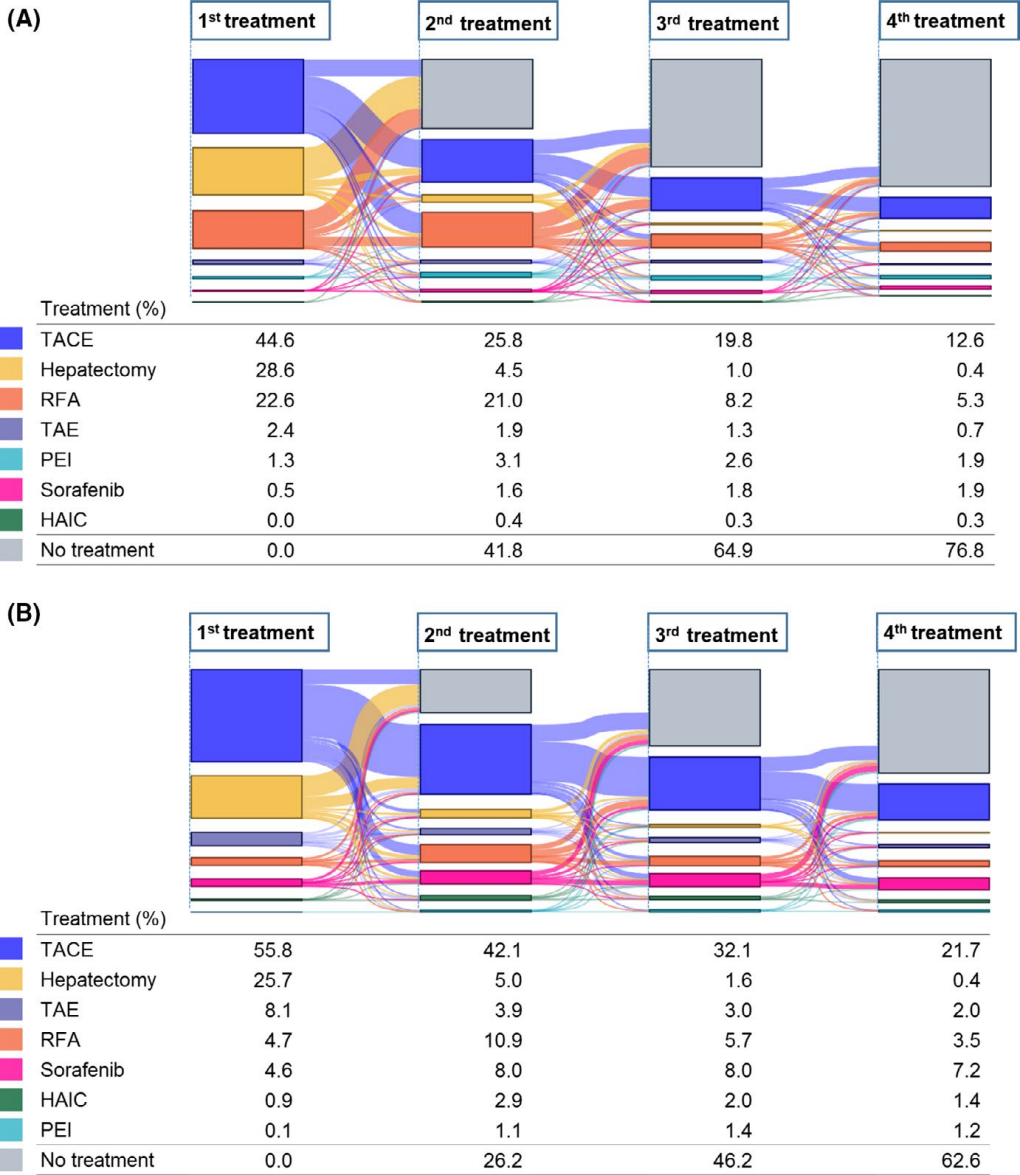
Sankey diagram of treatment flow for HCC patients. Change of treatments from first to fourth‐line were observed in early (A) and advanced (B) stages. The color indicates the treatment type and the flow size as percentages of patients. TACE, transcatheter arterial chemoembolization; TAE, transcatheter arterial embolization; RFA, radiofrequency ablation; PEI, percutaneous ethanol injection; HAIC, hepatic arterial infusion chemotherapy

Next, the treatment flow for HCC patients was analyzed by disease stages to provide an understanding of the complete picture of HCC patient treatment. To understand treatment flow, the first to fourth‐line treatments are illustrated in a Sankey diagram (Figure 1). The most frequent treatment was TACE therapy, followed by hepatectomy, for first‐line treatment in both early and advanced stages. TACE was most frequently used also in second, third, and fourth‐line treatments in both stages but the ratio of hepatectomy drastically decreased in the second line. Because the major reason of no treatment was recorded for recovery or remission of disease (data not shown), the increased ratio of no treatment in the later lines suggested the treatment success. The majority of patients treated with hepatectomy received no treatment in the later lines, while the majority of patients treated with TACE received TACE again, suggesting the higher success rate in hepatectomy than in TACE. The ratio of no treatment in the later lines was higher in early stages than in advanced stages.

Table 3 provides a more detailed analysis of HCC treatment choice for first‐line treatment. At all stages, the most common first‐line treatment was TACE (47.6%), followed by hepatectomy (27.8%) and RFA (17.9%). With tumor stage progression, the proportions of TACE, TAE, sorafenib, and HAIC increased, while those of hepatectomy, RFA, and PEI decreased.

**Table 3:** First‐line treatments for HCC patients

	All stages	Early stages	Advanced stages	*P*
	(*N* = 8999)	(*N* = 6594)	(*N* = 2405)	
TACE	47.6%	44.6%	55.8%	<0.001
Hepatectomy	27.8%	28.6%	25.7%	0.007
RFA	17.9%	22.6%	4.7%	<0.001
TAE	3.9%	2.4%	8.1%	<0.001
Sorafenib	1.6%	0.5%	4.6%	<0.001
PEI	1.0%	1.3%	0.1%	<0.001
HAIC	0.3%	0.0%	0.9%	<0.001

The differences in rates between early and advanced stages were assessed and *P*‐values were calculated using Fisher's exact test. HCC, hepatocellular carcinoma; TACE, transcatheter arterial chemoembolization; RFA, radiofrequency ablation; TAE, transcatheter arterial embolization; PEI, percutaneous ethanol injection; HAIC, hepatic arterial infusion chemotherapy.

Because TACE was the most common first‐line treatment, chemotherapeutic agents used for first‐line TACE were analyzed (Table 4). Epirubicin was used most frequently (44.1%), followed by miriplatin (23.6%) and cisplatin (12.3%). With stage progression, the proportions of epirubicin and miriplatin decreased and of cisplatin increased. Concomitant use of sorafenib for first TACE was reported in 3.2% of patients, and it was significantly higher in advanced stages than in early stages (Table 4).

**Table 4:** Chemotherapeutic agents and concomitant use of sorafenib for first‐line TACE

	All stages	Early stages	Advanced stages	*P*
	(N = 4283)	(N = 2940)	(N = 1343)	
Epirubicin	44.1%	45.3%	41.6%	0.026
Miriplatin	23.6%	24.6%	21.4%	0.020
Cisplatin	12.3%	11.1%	15.0%	<0.001
Epirubicin and mitomycin C	8.5%	8.5%	8.5%	1.000
Doxorubicin	2.9%	2.8%	3.3%	0.381
Doxorubicin and mitomycin C	1.9%	2.1%	1.3%	0.089
Cisplatin and miriplatin	1.8%	1.7%	2.0%	0.539
Concomitant use of sorafenib	3.2%	1.5%	6.9%	<0.001

The differences in mean values between early and advanced stages were assessed and *P*‐values were calculated using Fisher's exact test. TACE, transcatheter arterial chemoembolization.

The second‐line treatment chosen after the first TACE treatment was also analyzed (Table 5). Repeated TACE was conducted most frequently after the first TACE treatment in 44.2% of patients, followed by RFA for 22.9% in all stages. With tumor stage progression, the proportions of TACE and HAIC increased, while those of RFA and PEI decreased. The proportion of hepatectomy and TAE did not differ between stages. In early stages, 38.4% of HCC patients were treated with repeated TACE, followed by RFA in 28.2% and hepatectomy in 4.1%. In advanced stages, 56.9% of HCC patients were treated with repeated TACE, followed by RFA in 11.4% and hepatectomy in 3.7%.

**Table 5:** Second‐line treatment after first‐line TACE

	All stages	Early stages	Advanced stages	*P*
	(*N* = 4283)	(*N* = 2940)	(*N* = 1343)	
TACE	44.2%	38.4%	56.9%	<0.001
RFA	22.9%	28.2%	11.4%	<0.001
Hepatectomy	4.0%	4.1%	3.7%	0.613
TAE	2.3%	2.3%	2.2%	0.825
PEI	2.1%	2.4%	1.4%	0.029
HAIC	0.7%	0.2%	1.6%	<0.001

The differences in mean values between early and advanced stages were assessed and *P*‐values were calculated using Fisher's exact test. TACE, transcatheter arterial chemoembolization; RFA, radiofrequency ablation; TAE, transcatheter arterial embolization; PEI, percutaneous ethanol injection; HAIC, hepatic arterial infusion chemotherapy.

## 4 DISCUSSION

Detailed information on the baseline characteristics of 8999 Japanese HCC patients was presented by disease stage. The results suggest that HCC is a common disease in elderly persons in Japan. The observation period decreased with tumor stage progression, suggesting that HCC patients in advanced stages had a worse prognosis. Many patients had HCV hepatitis and concomitant hypertension, type 2 diabetes, cirrhosis, and hyperlipidemia. Interestingly, the rate of HCV hepatitis decreased in HCC patients with stage progression. In Japan, a long‐term HCC surveillance program contributed to a significant increase in overall survival.^5^ Therefore, HCC patients with HCV‐hepatitis may have more opportunities for HCC surveillance and start treatment at earlier stages than HCC patients with HBV‐hepatitis and nonviral hepatitis.

In this study, detailed information on treatment flow for the first‐line and subsequent treatments for HCC patients was also presented. TACE was the most common first to fourth‐line treatment in both early and advanced stages. The TACE treatment rationale is that the intraarterial infusion of cytotoxic agents followed by tumor‐feeding blood vessel embolization induces strong cytotoxic and ischemic effects in tumors. Database analyses in other countries also reported that TACE was the most frequently selected first‐line treatment.^14^, ^15^

The Sankey diagram in this study showed the complicated pattern of treatment flow for HCC patients. The ratio of treatment success, indicated by the ratio of no treatment in the later lines, was higher in early stages than advanced stages. The majority of patients treated with hepatectomy received no treatment in the later lines, while the majority of patients treated with TACE received TACE again. The choice of repeated TACE after the failure of first‐line TACE remains controversial, although this study indicated that repeated TACE was the most common treatment. Randomized, controlled clinical trials have established the survival benefits of TACE.^7^ Despite the benefits, however, TACE frequently causes hepatic decompensation. Of 102 HCC patients with Child‐Pugh A scores, 30.4% and 10.8% had Child‐Pugh B and C scores, respectively, 1 month after TACE treatment.^16^ Multivariate analysis showed that larger tumor size, higher serum AFP, and lower serum albumin at baseline were the predictors for hepatic decompensation. In the present analysis, Child Pugh scores were comparable between tumor stages, but ALP and γ‐GTP values were higher in advanced stages. The higher risk of TACE causing liver function to worsen in advanced stages should be considered in the choice of treatment.

As chemotherapeutic agents for TACE, epirubicin was used most frequently, followed by miriplatin and cisplatin. Although the choice of chemotherapeutic agents for TACE has not been standardized and remains inconclusive, it is important to note that the proportions of epirubicin and miriplatin decreased and of cisplatin increased with stage progression in this study. A phase 3 randomized trial of TACE treatment comparing epirubicin and miriplatin showed comparable impacts on overall survival and time to treatment failure.^17^ Previous reports comparing different chemotherapeutic regimens for TACE showed higher efficacy and a higher rate of adverse events in cisplatin‐treated patients than in those treated with epirubicin or miriplatin.^18^ The regimen with a higher expected efficacy may be preferred for advanced stages despite a higher risk of toxicity.

Concurrent use of sorafenib in first‐line TACE was more frequent in advanced stages in this study. Enhanced efficacy and prolonged tumor control using TACE are expected with concurrent use of sorafenib.^19^, ^20^ In addition, concurrent sorafenib therapy extends the interval to subsequent TACE.^21^ In a meta‐analysis of randomized studies, time to disease progression was significantly prolonged in the TACE‐sorafenib group compared with the TACE‐alone group.^22^, ^23^ Concomitant use of sorafenib for TACE may have a significantly increase the benefit of TACE treatment.

Database research using a medical claims database can provide reliable results, but there are some limitations in data interpretation. A limitation of database research is the difficulty in understanding the reasons for discontinuation or change in treatments, whether due to intolerable adverse events or insufficient efficacy. Laboratory values may provide information regarding potential toxicity or efficacy, but they are available in only a limited number of patients. Another limitation is that the database did not clearly show the type of TACE selected. Recently, new types of TACE including drug‐eluting beads ‐TACE have been widely adopted. The randomized, controlled trial of drug‐eluting beads‐TACE versus conventional TACE for HCC indicated that they were equally effective and safe.^24^

In conclusion, HCC patients' baseline characteristics and treatment flow differed between early and advanced stages. Continuous analysis of the database with longer follow‐up may provide useful information about treatment selection and prediction of outcome such as survival.

## CONFLICT OF INTEREST

All authors are employees of Eisai Co., Ltd., Tokyo, Japan.

## ETHICS STATEMENT

This study analyzed anonymized medical claims data, and there was no active enrollment or active follow‐up of study subjects, and no data were collected directly from individuals. Therefore, it was not necessary to receive ethics committee approval for this study.

## Data Availability

The claims database used for this study can only be obtained by purchasing from a vendor (Medical Data Vision Co., Ltd; [URL] http://www.mdv.co.jp/).
